# Suicide in Sri Lanka 1975–2012: age, period and cohort analysis of police and hospital data

**DOI:** 10.1186/1471-2458-14-839

**Published:** 2014-08-13

**Authors:** Duleeka W Knipe, Chris Metcalfe, Ravindra Fernando, Melissa Pearson, Flemming Konradsen, Michael Eddleston, David Gunnell

**Affiliations:** School of Social and Community Medicine, University of Bristol, Bristol, United Kingdom; South Asian Clinical Toxicology Research Collaboration (SACTRC), Faculty of Medicine, University of Peradeniya, Peradeniya, Sri Lanka; Department of Forensic Medicine and Toxicology, University of Colombo, Colombo, Sri Lanka; Department of International Health, Immunology and Microbiology, Faculty of Health Sciences, University of Copenhagen, Copenhagen, Denmark; Pharmacology, Toxicology & Therapeutics, University/BHF Centre for Cardiovascular Science University of Edinburgh, Edinburgh, UK

**Keywords:** Sri Lanka, Pesticide poisoning, Suicide, Period effects, Birth-cohort effects

## Abstract

**Background:**

Sri Lanka has experienced major changes in its suicide rates since the 1970s, and in 1995 it had one of the highest rates in the world. Subsequent reductions in Sri Lanka’s suicide rates have been attributed to the introduction of restrictions on the availability of highly toxic pesticides. We investigate these changes in suicide rates in relation to age, gender, method specific trends and birth-cohort and period effects, with the aim of informing preventative strategies.

**Methods:**

Secular trends of suicide in relation to age, sex, method, birth-cohort and period effects were investigated graphically using police data (1975–2012). Poisoning case-fatality was investigated using national hospital admission data (2004–2010).

**Results:**

There were marked changes to the age-, gender- and method-specific incidence of suicide over the study period. Year on year declines in rates began in 17–25 year olds in the early 1980s. Reduction in older age groups followed and falls in all age groups occurred after all class I (the most toxic) pesticides were banned. Distinct changes in the age/gender pattern of suicide are observed: in the 1980s suicide rates were highest in 21–35 year old men; by the 2000s, this pattern had reversed with a stepwise increase in male rates with increasing age. Throughout the study period female rates were highest in 17–25 year olds. There has been a rise in suicide by hanging, though this rise is relatively small in relation to the marked decline in self-poisoning deaths. The patterns of suicides are more consistent with a period rather than birth-cohort effect.

**Conclusions:**

The epidemiology of suicide in Sri Lanka has changed noticeably in the last 30 years. The introduction of pesticide regulations in Sri Lanka coincides with a reduction in suicide rates, with evidence of limited method substitution.

**Electronic supplementary material:**

The online version of this article (doi:10.1186/1471-2458-14-839) contains supplementary material, which is available to authorized users.

## Background

The epidemiology of suicide in low and middle income countries (LAMIC) in Asia differs from the west. In most western countries the incidence of suicide increases with age; in rural Asia there is also an earlier peak in incidence in 15–30 year olds [1–4]. A suggested reason for the high incidence in young people is that self-harm with low suicide intent is highest in 15–34 year olds worldwide and is often impulsive. In countries such as the UK, the methods that impulsive self-harmers have readily available, such as paracetamol, have low case-fatality (often less than 1%) [5]. In contrast, in rural Asia with the ready availability of highly toxic pesticides, some with case fatalities over 20% [6], impulsive acts more often result in completed suicide.

If the high rate of suicide in young people in rural Asia is a consequence of high lethality methods being used in impulsive acts of self-harm, reducing the toxicity of the pesticides available should reduce the case fatality associated with intentional poisoning and alter the epidemiology (age/sex patterns) of suicide. Consequently there should be a lowering of suicide incidence in young people, as well as a reduction in the overall suicide rate so long as equally lethal alternative methods are not adopted (method substitution).

Sri Lanka was notorious for having one of the highest suicide rates in the world in the early 1990s [7]. In subsequent years its suicide rate declined dramatically, a fall in rates which coincided with a series of bans on WHO class I pesticides starting in 1984 [8, 9]. In a previous analysis of suicides in Sri Lanka (1975–2005), we found evidence that reductions in suicide in both men and women coincided with the ban of the most toxic pesticides [8]. This decline in suicide rates did not appear to be associated with secular trends in other risk factors for suicide such as unemployment, alcohol misuse, divorce or Sri Lanka’s civil war (1983–2009). Previous investigations of the decline did not assess age, birth-cohort or period specific effects on suicide trends; such an analysis would help clarify the impact of regulations on particular age groups and possible long term effects on a generation of individuals exposed during their early life to a high incidence of suicide in their community. Furthermore, since our earlier analysis, a series of company trials and further regulations, starting in 2004 and culminating in 2008, have occurred in Sri Lanka, these resulted in reductions in the toxicity of highly toxic paraquat formulations. These interventions were followed by a complete ban on paraquat, dimethoate, and fenthion, from 2011 [9, 10].

Using police and hospital data we investigated: i) changes to age/gender and method specific pattern of suicide in Sri Lanka between 1975 and 2012; ii) changes in case fatality following pesticide regulations; and c) investigated the consequence of pesticide availability/toxicity on period and birth-cohort effects.

## Methods

### Context

Sri Lanka is an island state with a population of 20.3 million (2011 census) situated off the south east coast of India. 77% of Sri Lanka’s population lives in rural areas (2011 census) and nearly a third of those employed work in the agricultural sector [11].

### Suicide data

Age, gender and method specific suicide data were obtained, where available, for 1975–2012 from the Department of Police, Division of Statistics, Sri Lanka (http://www.police.lk/index.php/crime-trends). These publicly available data cover the majority of the island, but due to the civil war (1983–2009), data from three districts, Mullaitivu, Kilinochchi and Mannar, are missing from 1998–2004. We do not have the data broken down by district for the other years but assume they exclude these areas, because the years we have district level data confirm their exclusion during the civil war and we have no reason to suspect they would have been included for any other reason in the other years (post 1983). Based on the recent census (2011) these districts account for 1.5% of the total population and therefore their omission is unlikely to distort observations of the overall trends.

Suicide deaths were coded based on the suicide method used, into 1 of 8 categories between 1975 and 1996; 1 of 9 between 1997 and 2001; and 1 of 15 from 2002 onwards (Table 1). Using the approach employed by Gunnell *et al*. [8], we combined suicide deaths coded as ‘other’ and ‘all self-poisoning’ into a single self-poisoning category for all suicides prior to 2002 [8]. Method specific data was available from 1975–2012, however, data by age and gender is incomplete. Data is only available as follows: method and gender data for 1978, 1980 and 1982–2012; method, gender and age data for 1980–1985 and 1989–2012. As data are missing by age and gender for 3 years between 1985 and 1989, we only present age, gender and method specific data from 1989 onwards. Data were broken down into 5 year age bands, except for the two youngest and the oldest groups (8–16,17-20,21-25,26-30,31-35,36-40,41-45,46-50,51-55,56-60,61+). Data for 2007 were only available for 11 months, so figures were inflated by a factor of 12/11.

**Table 1:** **Categories of suicide deaths coded during different periods**

	Coding periods		
	1975-1996	1997-2001	2002-2010
Number of suicide methods/categories	8	9	15
Suicide methods/categories	Poisoning		
	Hanging	Hanging	Hanging
	Jumping in front of train	Jumping in front of train	Jumping in front of train
	Drowning	Drowning	Drowning
	Burning	Burning	Burning
	Shooting	Shooting	Shooting
	With sharp cutting instrument	With sharp cutting instrument	With sharp cutting instrument
	Other means	Other means	Other means
		Insecticide/Pesticide poisoning	Insecticide/Pesticide poisoning
		Acetic Acid Poisoning	
			Firearms
			Explosives
			Ingestion of acids
			Medicinal drug overdose
			Plant Poisoning
			Jumping from a height
			Ingestion/injection of addictive drugs

### Hospital data

Publicly available hospital admission and hospital death data for poisoning (ICD10 codes: T36-T65), by age and gender, were obtained from the Ministry of Health for Sri Lanka. Age and gender data was only available for 2004–2010. Data were available for the following age categories: 5–16, 17–49, 50–69 and 70 +.

### Population data

Censuses have been carried out in 1971, 1981, 2001 and 2011 in Sri Lanka. Whilst the final 2011 census results had not been released at the time of this analysis, data from a 5% sample of the census were available and we used this for relevant rate estimates. Mid-year population estimates for 1975–2010 were obtained from the Registrar General for Sri Lanka (publicly available). With regard to the estimates, there were three abrupt changes to the population estimates for different age groups centred on the three census years (1981, 2001 and 2011) (see Additional file 1), suggesting the mid-year population figures had been imperfectly estimated for the different age groups. To adjust for this, we recalculated the mid-year populations between the census years (1971–2011), for further details see Additional file 1. We used these estimates to calculate age and gender specific suicide rates (5 year age bands). The population estimate for 2012 has not been released; therefore the 2011 population was used as the denominator. Three year moving averages were used to smooth the estimates of trends in rates for graphical presentation, with averages centred on the middle year, e.g. the average rate for 1999–2001 would be plotted against 2000.

### Analysis

Graphical presentations were used to investigate secular trends of suicide in relation to age, sex, method, period and birth cohort. Age groups were collapsed into four broad age categories for ease of interpretation (17–25, 26–35, 36–55 and 55+). The hospital data was used to investigate trends in poisoning admissions, and case-fatality was calculated using information on live discharges and in-hospital deaths. Case fatality is reported as number of deaths per 100 admissions.

In order to investigate birth-cohort effects, we identified the birth years of the population represented in the suicide data available (1975–2012). We then tracked forward the age/gender specific suicide rates for each cohort. For example to calculate the 21–25 age-specific rate for the 1960 birth cohort, the mean suicide rate of the 21–25 age group over the years of 1981–1985 was used. Gender specific rates were plotted against 5-year age groups for each birth cohort.

We also investigated period effects by calculating the rates of suicide for each age group spanning five year periods starting from 1975. We did this by averaging the age/gender specific rate for each 5-year time period and plotted this against 5-year age groups for each calendar period.

In order to assess whether a period or cohort effect model was a better fit for our data we employed the Akaike information criterion (AIC) to help us assess the quality of the model for our data [12]. A smaller AIC score reflects a better fit. For each gender, two Poisson regression models were fitted. The first model regressed the number of suicide events against age and cohort group; this model had 25 parameters. The second regressed the number of suicide events against age and period group; this model had 16 parameters. The resulting log likelihood function for each model was used to calculate the AIC score (AIC = 2*parameters-2*log likelihood). We acknowledge that age, period and cohort effects are confounded, and that what appears to be an effect of one of these factors on rates, can always be explained as being the result of some combination of the other two [13]. However we are assuming that age and period (younger more impulsive; changing availability of highly toxic pesticides) are more parsimonious explanations of changing rates than cohort effects (coping strategies set by exposure to high rates at a sensitive age leads to higher risk of suicide later in life). We would only consider the changing rates as cohort effects if this model described the data in a more straightforward way. That is we would need an interaction between age and period to achieve the same fit to the data as a cohort effect model.

## Results

### Age-gender specific trends

**Figure 1:**
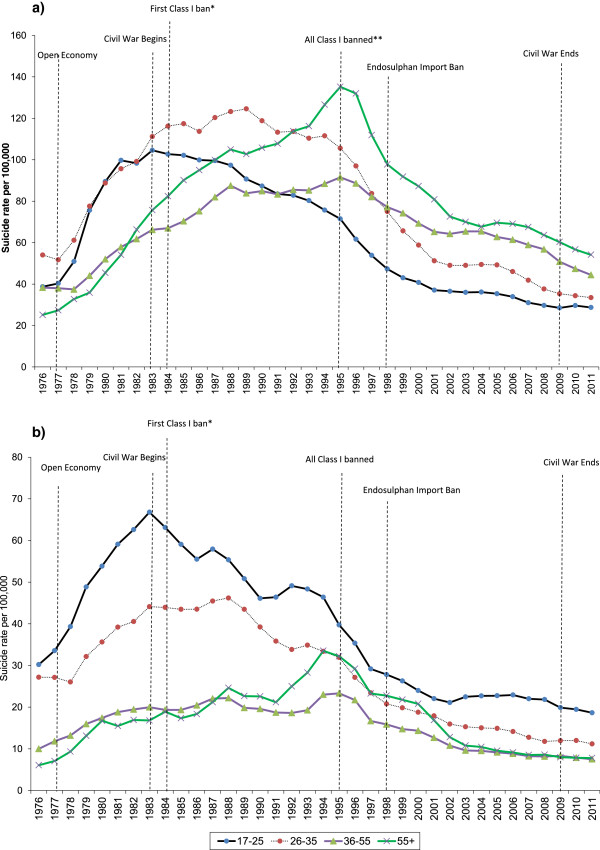
**Age-specific suicide rates in Sri Lanka (3 years moving averages) 1975–2012 (a) Male (b) Female.** Dotted vertical lines mark key historical milestones. *Class I Organophosphate - methylparathion and parathion. **All remaining Class I pesticides (monocrotphos and methamidophos).

In all age/sex groups suicide rates rose in the 1970s and early 1980s, before falling, with reductions starting earlier in the younger age groups. Age specific trends in the incidence of suicide differ between the sexes (Figure 1).

**Figure 2:**
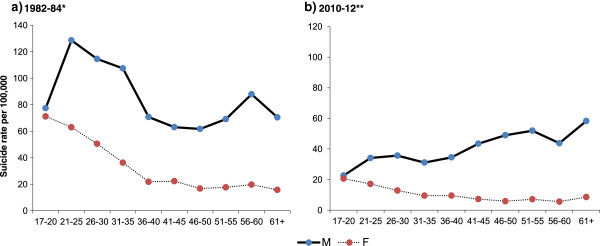
**Age profile of suicide by gender in (a) 1982–84 and (b) 2010–12.** *Denominator 1983 mid-year population, **Denominator 2011 census population (5% sample).

In males, up until 1986–88 the suicide rate in 17–25 year olds was higher than that in the older age groups (>35 year olds), likewise up until 1990–92 the incidence in 26–35 year olds was higher than that in the older age groups, but by 1991–93 the over 55 s surpassed all other ages, with a maximum rate of 135 per 100,000 in 1994–96. This was then followed by a rapid decline in rates, with more than a halving of all age-specific rates over a 10 year period, coinciding with the introduction (in 1995) of the last of a series of WHO class I pesticide bans which had started in 1984. The rates in the 17–25 and 26–35 year age groups, however, had already started to decline from the early 1980s and late 1980s respectively. The most marked decline in male suicide rates between 1994–96 and 2010–12 was in the 26–35 and 55+ age groups. Since 1999, the risk of suicide appears to increase with age, with a 2 fold difference in rates between 17–25 year olds and those aged 55 + .In contrast to males, younger females (<35 years) had the highest rate throughout the period. The incidence in 17–25 year olds reached a peak in 1982–84 (66 per 100,000) (Figure 1). Pre-1987 there were very wide (up to 5 fold) differences in rates between the youngest age group and the oldest, although subsequently these rates started to converge. Similar to the trends seen in men, the suicide rate in the 17–25 and 26–35 year olds started to decline before 1995. In the youngest age group (17–25), rates started to decline from the early 1980s and in the 26–35 age group the decline started in the late 1980s.Figure 2 shows age-specific suicide rates at two time points chosen to reflect the periods where data by age, gender and method were available, and when differences in rates between the youngest and oldest age groups were the largest (1982–84) and smallest (2010–12). In 1982–84 the highest rates in males were in the 21–25 age group. Rates fell with increasing age, except for a small rise in rates in 51–60 year olds relative to 36–50 year olds. By 2010–12 (Figure 2b) this pattern had reversed with linear increases in suicide rates with increasing age in men. In females, suicide risk in both 1982–4 and 2010–12 fell with increasing age, but the difference in rates between the youngest and oldest age groups was far greater in the 1980s compared to the 2000s.

### Method specific trends

**Figure 3:**
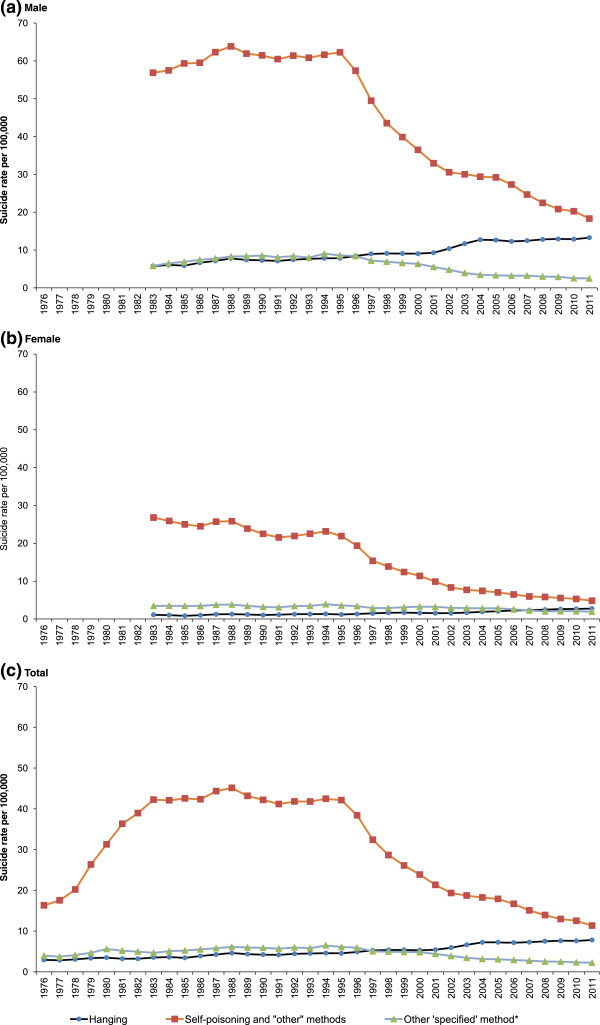
**Method specific suicide trends (3 year moving averages) (a) Male method specific suicide trends - 1982–2012 (b) Female method specific suicide trends - 1982–2012 (c) Overall method specific suicide trends - 1975–2012.** *Includes drowning, fire-arm, cutting/stabbing, burning, jumping in front of a moving vehicle and jumping from a height.

**Figure 4:**
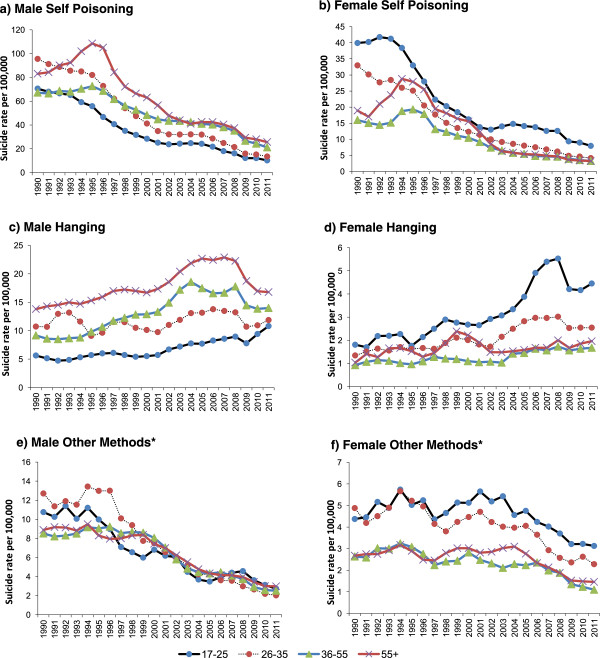
**Method, age and gender specific suicide trends 1989–2012 (3 year moving averages) - note that different scales are used on the y-axes for different methods (a) Male age-specific suicide rates for self-poisoning (b) Female age-specific suicide rates for self-poisoning (c) Male age-specific suicide rates for hanging (d) Female age-specific suicide rates for hanging (e) Male age-specific suicide rates for all other methods (f) Female age-specific suicide rates for all other methods.** *Includes drowning, fire-arm, cutting/stabbing, burning, jumping in front of a moving vehicle and jumping from a height.

Figure 3 shows method specific suicide rates for both genders (1975–2012) and for males and females separately (1982–2012). Method, age and gender specific suicide rates are available from 1989–2012 (Figure 4) during which time self-poisoning and hanging accounted for 88% of all suicides. Self-poisoning accounted for the majority of suicides, with a peak incidence of 42 per 100,000 in 1994–96 (79% of all suicides), falling to 11 per 100,000 by 2010–12 (48% of suicides) (Figure 3).The incidence of hanging increased from 3 per 100,000 in 1975–77 (13% of suicides) to 8 per 100,000 in 2010–12 (36% of suicides). Hanging rates have increased in both genders, and the incidence of hanging rises with age in men and decreases with age in women (Figure 4 - note that different scales are used on the y-axes for different methods). Between 1989–91 and 2010–12, the incidence of hanging in men and women increased most markedly in the youngest age group (17–25): from 6 to 11 per 100,000 in men and 2 to 4 per 100,000 in women. Whilst age and gender specific hanging rates have increased, this compensatory rise (possibly due to method “substitution”) is much lower than the overall decline of pesticide-related suicides.The rates of suicide by all methods other than poisoning and hanging remained fairly constant between 1980 and 2001 (Figure 3), with a slight decline from 2000. In men there was a decline of suicide by other methods from 1990 to 2001 for all age groups, whilst in women the rates remain fairly constant by age until mid-2000, at which point rates start to decline (Figure 4).

Additional file 2 shows age, gender and method-specific suicide rates in two time periods (1982–84 and 2010–12). The age and gender distribution of poisoning suicides in 1982–84 closely resembles the patterning seen for overall suicides (Figure 2). In 2010–12 poisoning suicides still closely mirror the overall suicide pattern for the same period; however, in men the pattern by age of hanging related suicides has changed to strongly resemble poisoning suicides.

### Hospital admissions and case fatality

Hospital admission data stratified by age and gender were only available from 2004 to 2010. Age groupings were too broad to observe any meaningful patterns and therefore are not presented. Poisoning admissions have increased; male rates rose from 328 per 100,000 in 2004 to 463 per 100,000 in 2010 and in females rates rose from 270 to 421 per 100,000. Pesticide poisoning was the most common method in men until 2007, when other poisoning admissions were the most common. In women prescription/medicinal drug poisoning was the most common method (see Additional file 3). Organophosphorus and carbamate insecticide poisoning accounted for 72-75% of all pesticide admissions. Pesticide related case fatality dropped from 9.7 per 100 admissions in 2004 to 4.6 in 2010 in men and from 4.1 to 2.0 in women. Case fatality for drugs and other poisoning remained fairly constant throughout the period (1.1 per 100 admissions in men and 0.4 in women).

### Birth-cohort/period effect

**Figure 5:**
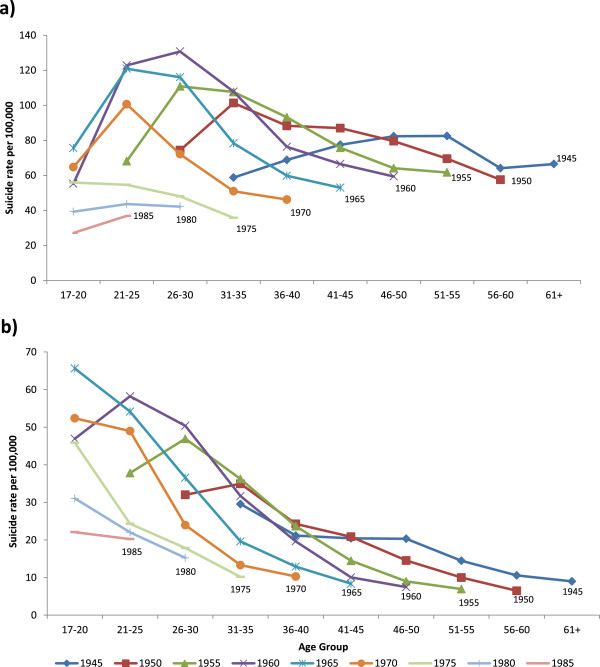
**Suicide deaths in 5 year successive birth-cohorts at different ages. (a)** Males; **(b)** Females.

**Figure 6:**
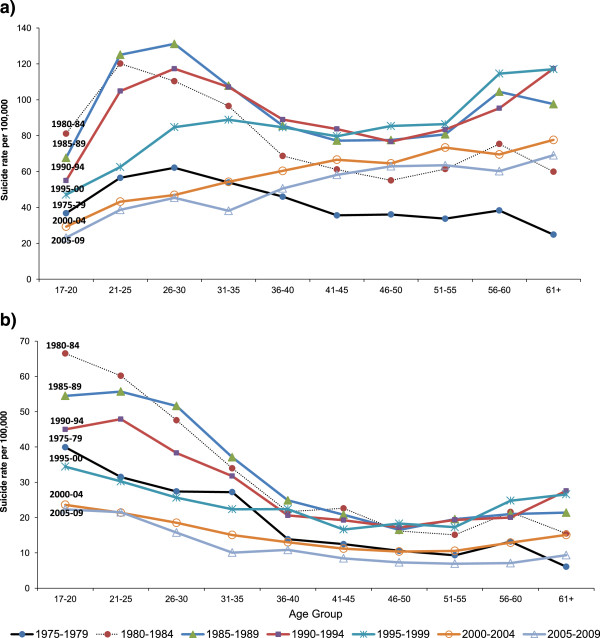
**Suicide deaths in 5 year periods at different ages. (a)** Males; **(b)** Females.

Figure 5 shows the suicide rate for each successive cohort from 1945 by age and gender. There is some evidence of a cohort effect with older cohorts having a higher suicide risk at all ages in both genders, except for the 1945 and 1950 birth-cohorts which have a lower rate than other cohorts for the <40 age groups. The 1945 cohort appears to have a different risk pattern to the other cohorts. In addition the cohorts born between 1950-1960, who experienced the ready availability of toxic pesticides during their young adulthood (17–30 years), appear to carry with them an elevated risk of suicide through life despite the introduction of pesticide regulations. When looking at birth-cohort effects by method (not shown), it appears to be self-poisoning suicides that underlies the pattern observed for overall suicide.Figure 6 shows the age-specific suicide rates for each successive period starting from 1975–1979. The suicide rates for the periods spanning 1980–1994 are higher than subsequent years in all age groups. With the exception of the oldest age group, suicide rates after 1995 were much lower than in previous years.

By calculating AIC for each birth-cohort and period effect model in men and women, we found that in men the AIC was 1698 for a birth-cohort model vs. 1620 for a period model, suggesting a slightly better fit for the latter models (smaller AIC score reflects a better fit). In women the difference was more pronounced, with an AIC score of 957 for the birth-cohort model vs. 672 for a period effect model.

## Discussion

Sri Lanka has seen dramatic changes to the age, gender and method specific pattern of suicide over the last 30 years; there are five main features to these changes. First, there have been year on year declines in all age groups since 1995; these declines began in the 17–25 year olds (in the early 1980s), followed by the 26–35 and ≥36 age groups in the late 1980s and mid 1990s respectively. Second, there have been marked changes in the age pattern of suicide in males; in the 1980s the highest rate was in 21–25 year olds and rates declined with increasing age, whereas the opposite pattern was seen in the 2000s with rates increasing with age. Third, the reductions in rates appear to be driven by a decline in self-poisoning suicides. There has been a rise in suicide by hanging, but this has been small in comparison with the fall in self-poisoning suicide. Fourth, there was a rise in drug related poisoning admissions, especially in women, and some evidence of reductions in case fatality associated with all poisoning admissions. Last, there was evidence of possible birth-cohort and period effects, though the analysis suggests a stronger period effect.

### Strengths and limitations

A particular strength of this study is the wealth of detailed age and method specific national suicide mortality data spanning over 35 years; this is unusual for a LAMIC. We were able to identify groups with high suicide incidence and emerging patterns of method specific suicide. We are the first to report on age and gender trends of suicide in Sri Lanka having adjusted for the imperfect estimations of different age and gender population figures (see Additional file 1). This adjustment explains why the trends reported in this study differ to those previously reported [1, 3]. To our knowledge, this is also the first study to investigate birth-cohort and period effects in a LAMIC in Asia.

This evidence, however, should be considered in light of several limitations. One limitation is the incomplete coverage of suicide statistics from the Northern part of the country which was heavily affected by the civil war. Only 1.5% of the population, however, live in this area, so the overall findings of this study are unlikely to be greatly affected by their exclusion. A common problem of suicide statistics originating from LAMIC are their reliability in capturing true incidence. Systematic investigation of the quality and reliability of Sri Lanka’s suicide data has not been carried out. Anecdotally, from our fieldwork studies, we are aware that data quality and completeness may vary between police departments, but we have no evidence that data quality has changed over time in such a way as to bias our estimates of temporal trends.

The decriminalisation of suicide in 1998 in Sri Lanka coupled with changing attitudes towards suicide may result in more deaths being attributed to suicide post 1998, so we may have underestimated the extent of the recent reductions in suicide. Equally, it is possible that the suicide rate in women, especially young women, is underestimated. The act of suicide may jeopardise the family’s honour and social standing within a community [14] and therefore families may hide details regarding the incident or persuade doctors and coroners not to document the suicide and report it as an accident. This is likely to have affected the rate of suicide throughout the study period and is therefore unlikely to have influenced secular trends observed. In addition we were unable to investigate the emergence of method specific suicides by urban and rural areas separately. This information would have been useful in identifying any differential impact of pesticide regulations in the areas of the country where they are most often used and so most easily accessible. We have not explicitly investigated the impact of changes in Sri Lanka’s political and economic environment on suicide trends; our previous analysis indicated that these were unlikely to have had a major influence on recent trends.

Data on hospital admissions for poisoning are collected from all peripheral (community centres) and tertiary (teaching and special) hospitals in Sri Lanka. This data is collected by the Ministry of Health using a standard returns form from all hospitals. Due to limited facilities in peripheral hospitals, poisoning patients are often transferred to tertiary hospitals (47.2% of all poisoning admissions) [15]. With no linking of patient data for transfers, a degree of double counting of admissions occurs; which means we are likely to underestimate case fatality estimates, as some cases of self-poisoning as the denominator for our case fatality will be over-estimated as some individuals are included twice [16]. This will not have affected our findings in relation to case fatality trends, as no national changes have been made to hospital data reporting during the study period. Lastly, age- and sex-specific hospital admission data was only available for a relatively short time period, meaning we could not evaluate long term trends.

### Findings in context of literature

#### Asian suicide rates

In contrast to Sri Lanka’s declining suicide rate over the last 18 years, its neighbour India had fairly stable rates [2, 17], whilst in China rates have only declined markedly over the last 10 years [18, 19]. Both China and India can be considered to be similar to Sri Lanka as they both have a large proportion of their population engaged in agriculture. In other Asian nations such as Korea, Taiwan and Japan, pesticide poisoning accounts for a smaller proportion of total suicides (26% [20], 12% [21] and <4% [20] respectively). Furthermore, these countries are more economically advanced and have fewer agricultural worker (and therefore less easy access to, and familiarity with, pesticides) than is the case in Sri Lanka. Differences in the predominant religions and political situation may further compromise cross-country comparison in Asia.

As in Sri Lanka, the age and gender profile of suicide in China has changed [18, 22–24] in recent years. In the early 1980s in Sri Lanka, there were two peaks in the age-specific incidence of suicide in males, one in young people and the other in the elderly, similar to the pattern seen in China in the late 80s [24]. In more recent years, there is a linear increase in male rates in Sri Lanka and China with age - a pattern typical of that seen in many western countries [2, 18]. This linear pattern is also now seen in female rates in China [18]. Whilst female suicides in Sri Lanka have fallen over time, the age pattern – linear declines in rates with age are in marked contrast to those seen in the West (and China) where incidence is higher amongst older women. These changes to the age and gender profile in China and Sri Lanka, however, happened at different times. In China the changes started in the mid-late 90s, whereas in Sri Lanka these changes started in the early 80s. National data from India for the same time period are not available; therefore we are unable to make this comparison. Self-poisoning remains the commonest method in Sri Lanka, India and China, followed by hanging and drowning [2, 25].

#### Method substitution

Restriction of one method of suicide may lead to rises in the use of other methods for suicide (substitution). A review of suicide prevention through means restriction concluded that restriction leads to limited method substitution [26]. The extent to which this occurs, and its impact on overall suicide rates will depend on the lethality of the method restricted, the lethality of the individual’s alternative choice of method and the individual’s suicidal intent [27, 28]. For example in England and Wales the detoxification of domestic gas supplies saw a reduction in domestic gas poisoning suicides and a rise in drug overdose deaths [29]. This substitution effect, however, was not observed in all age and gender groups and was most apparent in women and younger men. This is consistent with our analysis of Sri Lankan data where restriction of pesticides appeared to result in a differential increase in hanging suicides by age and gender. Our data suggests that a degree of method substitution has occurred but because this rise in hanging was small in relation to the fall in self-poisoning suicides, the net effect was a marked reduction in overall suicide rates.

#### Birth-cohort and period effects

Investigations into birth-cohort and period effects have mainly been conducted in non-Asian countries [30–40], with the exception of studies conducted in Japan [41] and South Korea [42]. The majority of these studies present evidence of both period and birth-cohort effects [35–41], which is consistent with the findings from this study. The Sri Lankan evidence, however, points to a slightly stronger period effect which appears to relate to the availability of the most toxic pesticides in the market place. This is similar to the findings of three other studies conducted in the United Kingdom [40], Australia [33] and New Zealand [39]. These studies suggest a possible period effect as a result of changes in the availability of toxic domestic gas in the United Kingdom and barbiturates in New Zealand and Australia. In addition, whilst there will be combinations of age and cohort effects which will be equivalent this period effect is a more parsimonious explanation of the observed data.

### Interpretation and implications

Our data suggests that the introduction of bans on all WHO Class I pesticides have contributed to the reduction of overall suicide rates, age specific rates and poisoning-related case fatality. Reductions to case fatality may also be a result of better management of poisoning cases in hospitals [43]. These reasons, however, are unlikely to account for all the reduction in incidence. The time trends analysis suggests that there may be other important contributors. In 1970 and 1976, Endrin, toxaphene, chlordimeform, thallium and DDT were banned. These pesticide regulations had little effect on the overall and age-gender specific suicide rates. The limited effect may be because these pesticides were not commonly used/used for suicide; or that these pesticides were quickly replaced by other toxic formulations especially given the increase of pesticides available in the country post-open economy (1977).

In 17–25 men and 26–35 women, some plateauing of suicide rates occurred prior to the introduction of the bans on WHO class I pesticides. Also prior to pesticide regulations there were reductions in the incidence of suicide in 17–25 women. In 1980, when the plateauing and reductions started in these age groups, the ratification of the Control of Pesticides Act occurred, as did the establishment of both local and international knowledge and influence networks [9]. Whilst the Office of the Registrar of Pesticides was not established until 1983 and the WHO class I pesticide bans were not enforced until 1984, the establishment of local and international networks is likely to have increased awareness of the problem of pesticide related suicides. This may have resulted in unofficial changes to the control, sale and storage of pesticides.

Sri Lanka also experienced a long civil war which started in 1983 and ended in 2009. It is possible that the war impacted on the suicide rate, but this is unlikely, especially as the suicide rate increases pre-1983 and starts its decline 14 years before the end of the civil war.

In women there may also be an alternative explanation to the plateauing and reductions. Changes to the country’s economic situation in the late 1970s to early 90s saw a growing number of young women migrating to foreign countries and urban centres for employment [44]. Migration of these young women to foreign countries would have resulted in the removal of some at-risk individuals from the suicide statistics reported by the police, but would have been included in the census population figures. Migration of young women to urban centres where pesticide availability is less may also explain the earlier decline in the suicide rate in this group. Migration also allows these women to gain autonomy and control over their finances, which may in turn reduce the risk of self-harm.

Older men had the highest sex-specific rates of suicide in the post-regulation period. This pattern is in keeping with that seen in many western countries and may reflect increased levels of psychiatric morbidity and physical ill-health with age. It may also be that the years of poverty/stress accumulate as these men get older and increase suicide risk. It is also possible that as ageing is often associated with losses in income and occupation, especially in South Asian countries [45], older men experience a loss of status; this loss of status may then in turn increase their risk of suicide [46].

Pesticide regulations do not appear to have changed the pattern of suicide in women to more closely resemble western counterparts; a higher rate in young compared to older women prevails, whereas in the west older women have the higher rate. This may be viewed as contradicting the hypothesis that the high rate of suicide in young women is due to impulsive acts of self-harm being transformed into completed suicide due to the toxic pesticides employed. It would be expected therefore that the removal of these pesticides should see a reversal in the age profile to resemble a western profile. A possible reason to why we don’t see this reversal is that the higher suicide rate in these young women may reflect the high toxicity of the remaining pesticides. Young women, however, also have higher rates of suicide by other methods (e.g. hanging), which suggests that considerations to other sociocultural factors may also be important in this group. For example subsets of these young women are likely to be newly married and typically are newly residing with their husband’s family, sometimes at great distance from their own family. This environment gives rise to strained family relationships; in addition with economic and social changes, more young women are entering the work force, both locally and internationally. This group of women are threatening the conventional gender and generational hierarchy and this often leads to family arguments due to shifts in power [46, 47]. Attempts to address these issues may help to reduce the elevated rate seen in this group.

Sri Lanka’s secular trends suggest the existence of a period effect, especially in women, based on the availability of highly toxic pesticide. This is more parsimonious than a cohort effect, based on learning a style of coping due to being exposed to a high suicide rate at a sensitive age.

## Conclusion

The introduction of pesticide bans in Sri Lanka appears to have contributed to the dramatic reduction in suicide rates in young men and older women, albeit with evidence of limited method substitution. Other countries where pesticide related suicide are a major problem should follow Sri Lanka’s example by restricting access to the most toxic pesticides. Unlike in many other countries, young women in Sri Lanka have the highest rates of suicide amongst women suggesting that method restriction alone may not be adequate in reducing the higher rates in this group. Further work investigating specific risk factors in this group is needed to help inform future preventative strategies.

## Electronic supplementary material

Additional file 1: **Description of methods used to correct for sudden changes in census population figures.** Supplementary figures show the sudden changes graphically by gender. (PDF 285 KB)

Additional file 2: **Figures showing the age profile of suicide by gender and method.** (PDF 184 KB)

Additional file 3: **Figures showing hospital poisoning admission for the years 2004–2010 by gender.** (PDF 169 KB)

## Abbreviations

LAMIC: Low and middle income countries
AIC: Akaike information criterion.
