# Supplementary material for: Suicide in Sri Lanka 1975–2012: age, period and cohort analysis of police and hospital data
Source: BMC Public Health. 2014 Aug 13;14:839. doi: 10.1186/1471-2458-14-839 (PMC4148962; doi:10.1186/1471-2458-14-839)
Supplement: Supplementary file 1 — Additional file 1: Description of methods used to correct for sudden changes in census population figures. Supplementary figures show the sudden changes graphically by gender. (PDF 285 KB) [file 12889_2014_6975_MOESM1_ESM.pdf]

Supplementary Methods

Recalculating mid-year population estimates

The mid-year population estimates available from the Census Department by age and gender change suddenly centred on the census years (1981, 2001 and 2011). In order to correct for the sudden increases and decreases, we recalculated the mid-year estimates by calculating the population change for each age group by gender between census years (1971, 1981, 2001 and 2011). We then divided this by the number of intervening years between census years (10 years – 1971-1981; 20 years 1981-2001; 10 years 2001-2011). This factor increase/decrease was used to recalculate mid-year population estimates through interpolation.

Supplementary Figures

Supplementary figure 1 – Census Department male mid-year population estimates by age group

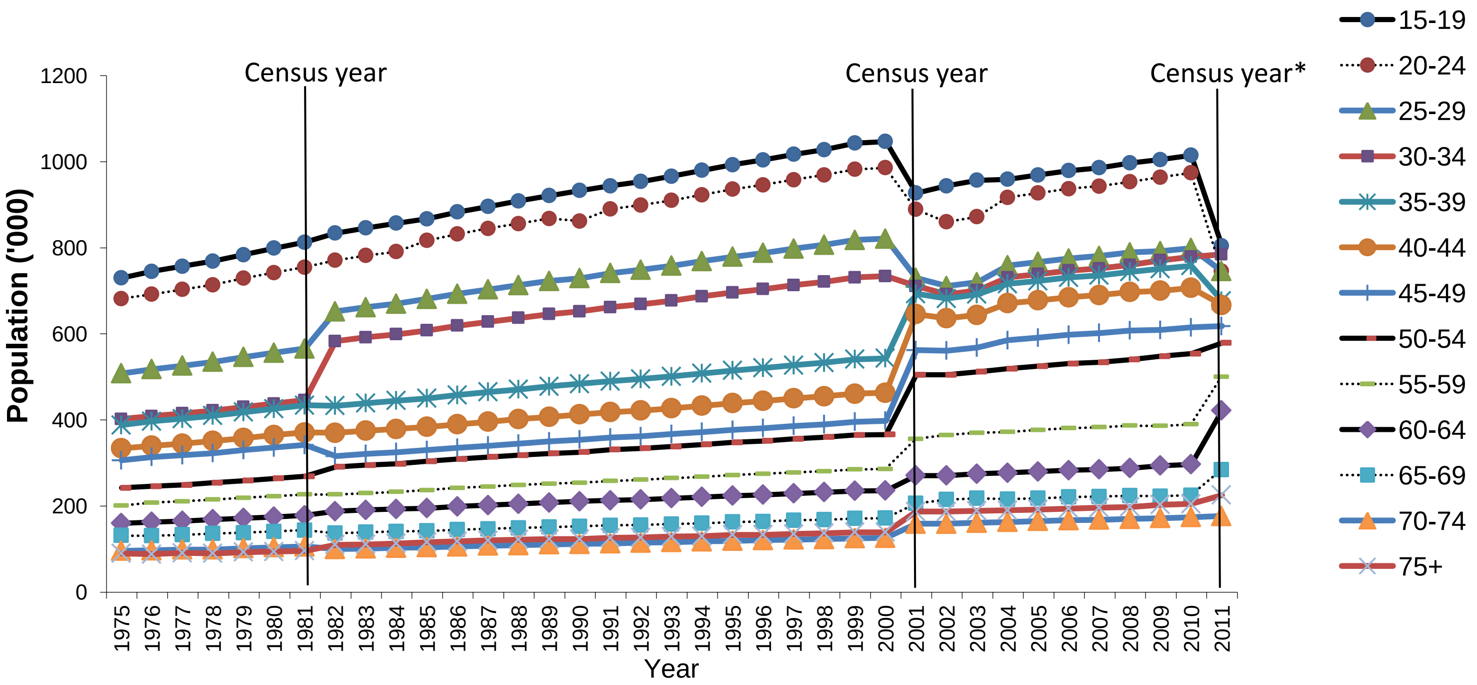

* Based on a 5% sample of the full census

Supplementary Figure 2 – Census Department female mid-year population estimated by age group

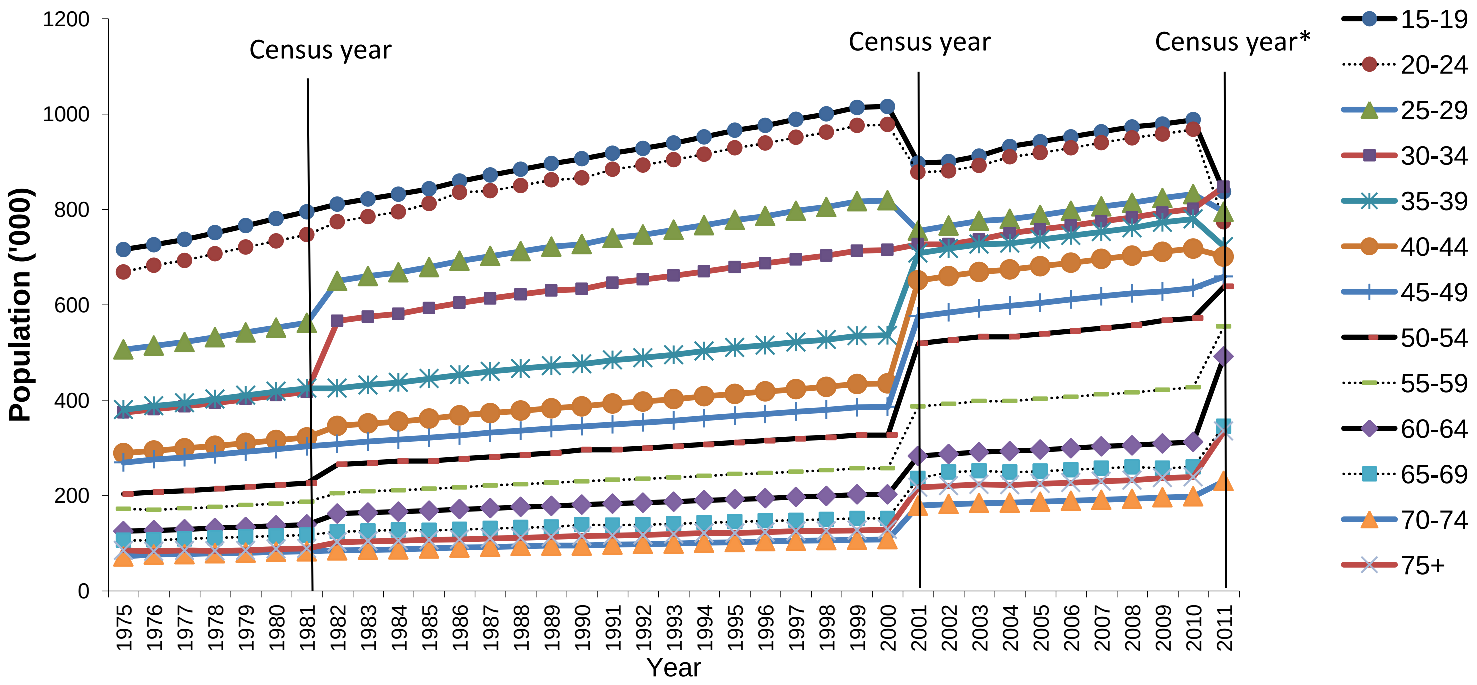

* Based on a 5% sample of the full census
