# Supplementary material for: Suicide in Sri Lanka 1975–2012: age, period and cohort analysis of police and hospital data
Source: BMC Public Health. 2014 Aug 13;14:839. doi: 10.1186/1471-2458-14-839 (PMC4148962; doi:10.1186/1471-2458-14-839)
Supplement: Supplementary file 2 — Additional file 2: Figures showing the age profile of suicide by gender and method. (PDF 184 KB) [file 12889_2014_6975_MOESM2_ESM.pdf]

Supplementary figure 3 – Age profile of suicide by gender and method

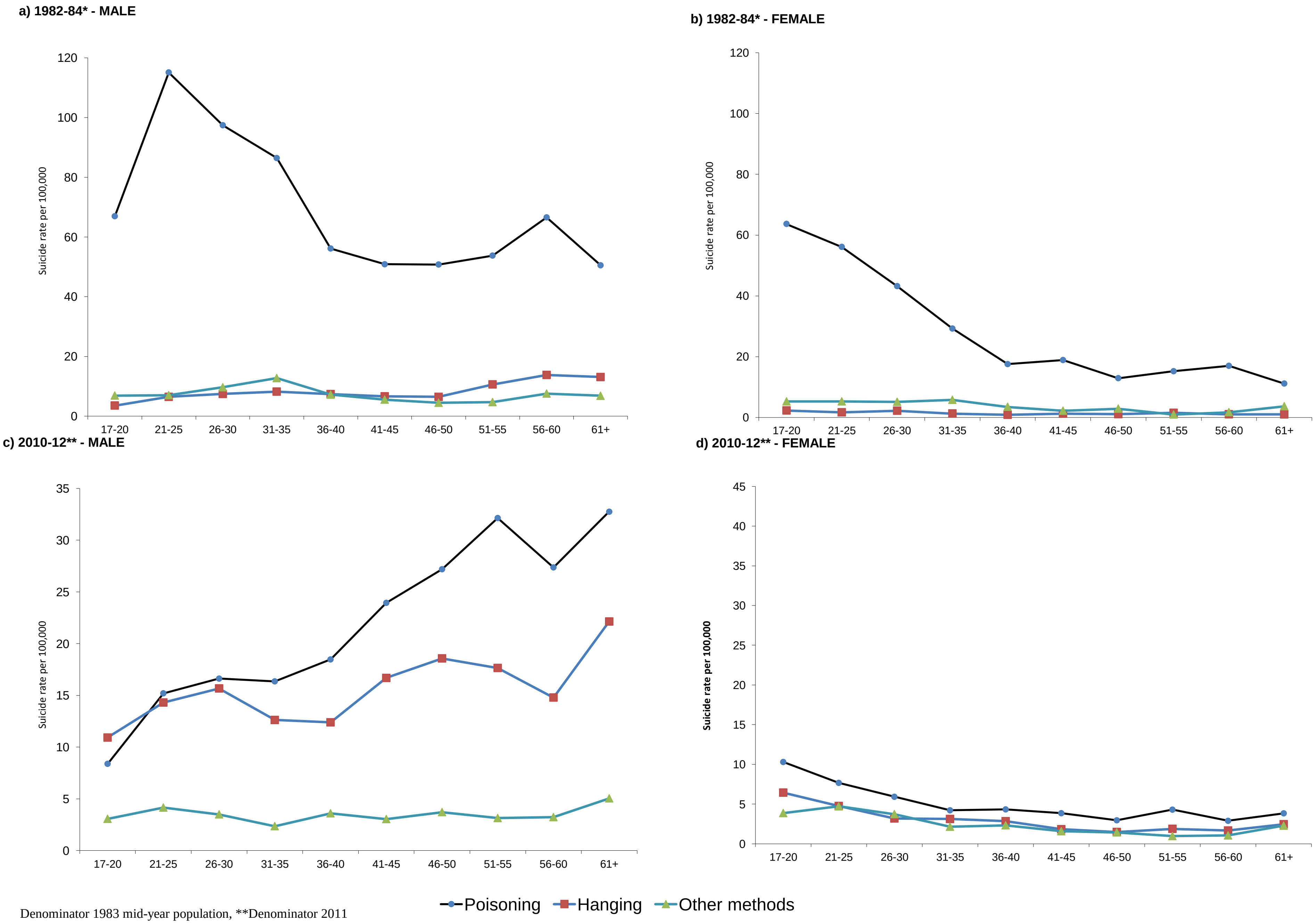

Denominator 1983 mid-year population, **Denominator 2011
