# Supplementary material for: Suicide in Sri Lanka 1975–2012: age, period and cohort analysis of police and hospital data
Source: BMC Public Health. 2014 Aug 13;14:839. doi: 10.1186/1471-2458-14-839 (PMC4148962; doi:10.1186/1471-2458-14-839)

Supplementary figure 4 – Hospital Poisoning admissions by gender (2004-2010)

a) Male

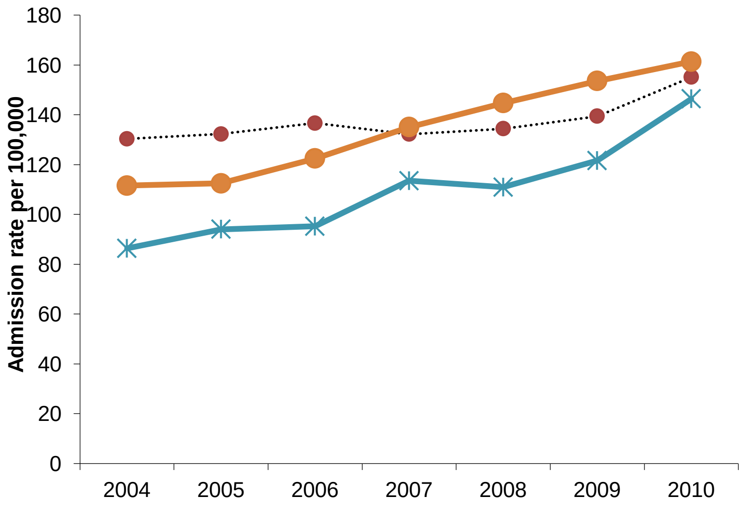

b) Female

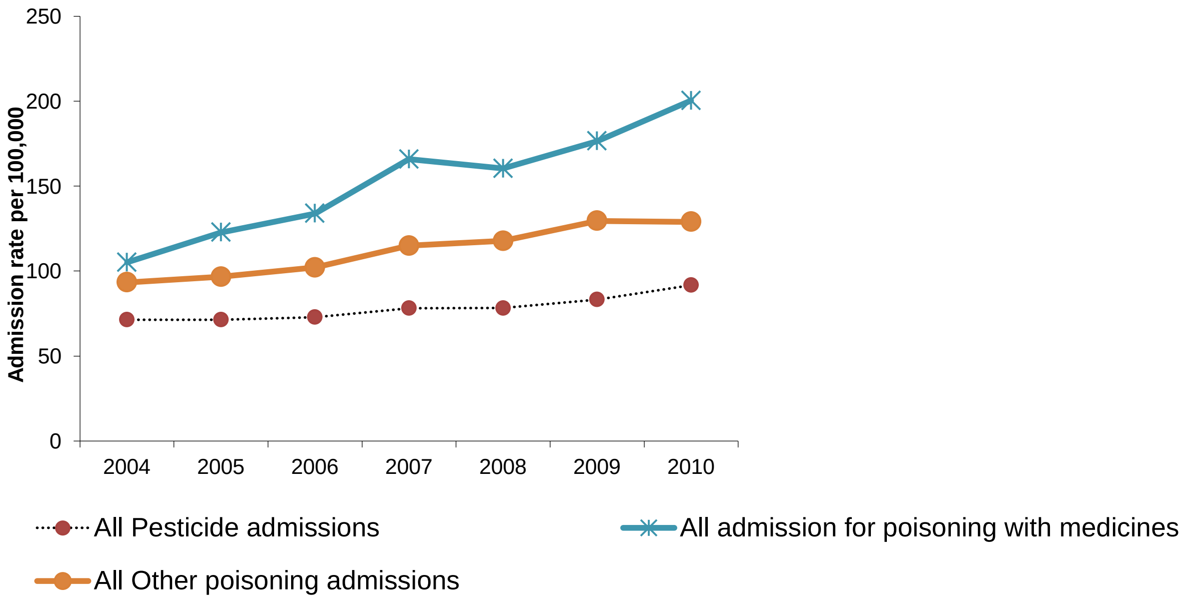

Supplement: Supplementary file 3 — Additional file 3: Figures showing hospital poisoning admission for the years 2004–2010 by gender. (PDF 169 KB) [file 12889_2014_6975_MOESM3_ESM.pdf]
